# The Excitations and Suppressions of the Times: Locating the Emotions in the Liver in Modern Chinese Medicine

**DOI:** 10.1007/s11013-012-9289-4

**Published:** 2013-01-31

**Authors:** Eric I. Karchmer

**Affiliations:** Appalachian State University, Boone, NC USA

**Keywords:** Chinese medicine, Depression, Constraint, Empiricism, Medical integration

## Abstract

This paper explores how doctors of Chinese medicine have borrowed from a long history of scholarship on the problem of “constraint” to develop treatments for modern emotion-related disorders, such as depression. I argue that this combining of medical practices was made possible by a complex sequence of events. First, doctors in the 1920 and 1930s were engaged in a critical reexamination of the entire corpus of Chinese medical knowledge. Spurred by the encounter with European imperialism, the sudden rise of Japan as a new power in East Asia, and the political struggles to establish a Chinese nation state, these scholars were among the first to speculate on the possible relationship between Chinese medicine and Western medicine. Second, in the 1950 and 1960s, doctors like other intellectuals were focused on national reunification and institution building. They rejected some of the experimental claims of their predecessors to focus on identifying the key characteristics of Chinese medicine, such as the methodology of “pattern recognition and treatment determination *bianzheng lunzhi*.” The flexibility of the new *bianzheng lunzhi* paradigm allowed doctors to quietly adopt innovations from their early twentieth century counterparts that they ostensibly rejected, ultimately paving the way for contemporary treatments of depression.

## Introduction

How do doctors of Chinese medicine treat a psychiatric condition, such as depression, when there is no such disease category in their medical practice? Doctors and scholars assert that Chinese medicine and biomedicine (or Western medicine, as it is known in China) differ considerably in almost all respects (Kaptchuk 2000; Shapiro 2003; Ren Yingqiu 任应秋 1957).^1^ As we will see in the discussion below, whether it is the views on the inner structure of the body, its principles of operation, the nature of human illness, or the strategies and techniques for medical intervention, these differences are quite striking. Given the epistemological and practical divide that separates them, developing a therapy in one medical system for a diagnosis in another would seem to be a daunting task. In the case of depression, this blending of medical systems is perhaps made even harder by the fact that Chinese medicine has only a very weak notion of the “mind,” the site where psychiatric disorders are thought to be located (see Farquhar 1998).

The anthropologist Zhang Yanhua gives us much insight into how a doctor of Chinese medicine might treat depression. In her book, *Transforming Emotions with Chinese Medicine*, she shows that doctors have developed an array of Chinese medicine treatments for emotion-related disorders 情志病. These types of illness are broadly considered to be a problem of constraint 鬱, which is both a pathological concept, describing obstructions to the normal functioning of the human body, and a *bing* 病, a nosological category that loosely resembles the biomedical concept of disease.^2^ There is not just one treatment for patients with constraint illness. Unlike biomedicine, where disease is the key rubric of diagnosis and therefore determines therapy, a Chinese medicine *bing* is often irrelevant for determining treatment, at least in contemporary practice. It is the concept of *zheng* or “pattern,” a subcategorization of a *bing*, that is the linchpin of diagnosis and an essential guide to therapy. In the case of constraint, Zhang Yanhua observed doctors diagnosing six different patterns, each pattern defined by a unique cluster of signs and symptoms. As she shows, once the appropriate pattern is determined, a basic formula is indicated. Formulas are herbal combinations, typically involving a half-dozen herbs or more. After determining a pattern, doctors will further refine their assessment, modifying the formula, adding or subtracting herbs, to produce an individualized treatment for each patient. This methodology of first identifying a broad *bing* category, then subdividing it into multiple patterns *zheng* and ultimately creating an individualized treatment for the patient is not unique to the treatment of emotion-related disorders. Known as *bianzheng lunzhi,* 辨證論治 “pattern recognition and treatment determination,” this methodology is considered the basic clinical methodology of Chinese medicine, one of the most fundamental and distinctive characteristics of Chinese medicine.

Zhang Yanhua does not explicitly state whether these treatments would be appropriate for depression. Indeed, she carefully avoids imposing any biomedical categories on the clinical work she observes. But, her caution about confusing categories does not prevent doctors from muddying the waters themselves. The willingness, even enthusiasm, of doctors to mix Chinese medicine with Western medicine is, in my opinion, one of the defining characteristics of contemporary Chinese medicine (Karchmer 2010). Although Zhang did not address the role Western medicine played in the clinical cases she observed, it is highly unlikely that biomedical concepts, disease categories, modern examination technologies, and pharmaceutical therapies were not at least on the doctors’ minds. This mixing of the two medical systems is particularly striking as one moves out of the outpatient clinics, where Zhang did her fieldwork, to the inpatient wards of Chinese hospitals. As I discovered as a student at the Beijing University of Chinese medicine in the late 1990s, doctors are required to make a “double diagnosis 双重诊断” for all patients admitted to a Chinese medicine hospitals.^3^ Although official hospital policy tries to minimize the use of Western medicine therapies, other social pressures push doctors, particularly on the inpatient wards, to rely on Western medicine therapies as much as, if not more, than Chinese medicine therapies. For example, economic reforms in the 1990s made hospitals responsible for their own bottom line, and prescribing expensive, new biomedical drugs became one important way for hospitals to become financially self-sufficient. These economic pressures may have been one consideration, among others, when I observed my clinical teachers prescribing Prozac for their depressed patients for the first time in 1999.

By taking an historical approach to this topic, I hope to provide an explanation for how some of the Chinese medicine therapies described in Zhang’s work have become important treatments for the biomedical condition of depression. I will argue that current treatment protocols have resulted from the articulation of long historical trends as described by Volker Scheid with rapidly changing social and political conditions in China during the early and mid twentieth century (Scheid 2013). This history is defined by two key moments. The first takes place in the 1920 and 1930s, when leading scholars of Chinese medicine were engaged in a critical and experimental reexamination of the entire corpus of traditional medical knowledge. Spurred by the encounter with European imperialism, the sudden rise of Japan as a new power in East Asia, and the political struggles to establish a Chinese nation state, these scholars were among the first to speculate on the possible relationship between Chinese medicine and Western medicine. The second stage occurs in the 1950 and 1960s, in a period of national reunification and institution building. Scholars were focused on identifying the key characteristics of Chinese medicine and promoting the methodology of *bianzheng lunzhi*. These two moments, the first defined by criticism and experimentation, the second by unity and consensus, ultimately laid the foundation for contemporary Chinese medicine treatments of depression.

## Patterns of depression

One interesting phenomenon regarding Chinese medicine “patterns *zheng*” of disharmony associated with depression is that they are frequently related to the Liver; not the anatomical organ, but the Chinese medicine organ that “stores blood and disperses *qi*.”^4^ Although Zhang Yanhua’s observations are not specific to depression, her findings help illustrate this point. In 150 cases of emotion-related disorders of constraint, she finds that 41 % of patients were diagnosed with one of three patterns of Liver *qi* stagnation 肝氣鬱結: Liver *qi* stagnation, Liver *qi* stagnation with fire, and (Liver) *qi* stagnation with phlegm congestion. About 50 % of the patients were afflicted with one of three patterns of deficiency, involving the Heart, Spleen, or Kidney: Heart yin deficiency, Spleen and Heart deficiency, and (Kidney) yin deficiency with fire (Zhang 2007, pp. 90–104).^5^ When we examine contemporary texts on Chinese medicine treatments for depression, we can find a similar primacy of the Liver. For example, in 2006 Zhou Ling conducted a study 408 patients diagnosed with depression. This researcher categorized her subjects according to six different patterns. About 70 % of the patients were classified as having one of four patterns involving some form of Liver *qi* stagnation. Another 70 % of patients, some overlapping with individuals in the first group, were categorized into four patterns of various Heart related pathologies (see Cao Xindong 曹欣冬 and Wang Wei 王伟 2009, pp. 47–48). Another author, Tang Qisheng proposes that depression should be subdivided into nine patterns, of which four are related to the Liver and three involve Liver *qi* stagnation specifically (Tang Qisheng 唐启盛 2006, pp. 217–228).

Pattern recognition is an interpretive process, so it is not surprising that individual doctors and researchers do not agree completely on the specific patterns associated with a *bing* or disease. But there is nonetheless a clear consensus that Liver *qi* stagnation is an important—if not the most important—component of emotion-related disorders and depression. This phenomenon is even more striking when we compare it to the history of constraint and, more recently, depression in Korea and Japan. As Soyoung Suh has demonstrated, the Liver and Liver-related therapies have not historically been important to the treatment of constraint in Korea. Researchers have begun to explore the role of the Liver in the depressive-like condition of *hwa*-*byung*, but this approach has been heavily influenced by the *bianzheng lunzhi* paradigm from mainland China (Suh 2013). The research of Keiko Daidoji provides an interesting foil to medical discourse in China. Although Edo and modern period Japanese physicians also located constraint illness in the Liver, they developed unique interpretations of role of the Liver in this illness and often deployed formulas that Chinese doctors would not consider appropriate for Liver pathologies (Daidoji 2013).

The significance of the Liver in contemporary Chinese medicine derives in part from the late imperial synthesis of medicine that developed in the Jiangnan region of China. As Volker Scheid has shown, doctors from this region gradually began to assert that the cause of constraint was internal, emotional factors and the location of pathology was increasingly in the Liver (Scheid 2013). As we will see below, it was also doctors from this region who exerted a decisive influence on the development of medicine in the twentieth century, pushing medical discourse in new directions. During the Republican era (1911–1949), a small but influential group of doctors from Jiangnan became involved in a far-reaching, critical reassessment of Chinese medicine, rejecting much of what had become standard medical practice in the region. These reformers were driven in part by the revolutionary spirit of this period. But, they were influenced most directly by the empiricist ethics that emerged out of the confluence of Qing evidential scholarship, the recent arrival of Western medicine and the discovery of Japanese Kampo medicine. During the Communist Era (1949—present), the influence of these elite reformers waned as a new generation of physicians came of age. After decades of political strife and war, national reconstruction and the recognition of the growing strength of Western medicine profession pushed this new cohort of leading physicians to shun self-critique, seek theoretical consensus, and create a “national medicine”. The institutionalized practice of Chinese medicine in the Communist period carried forward many of the experimental practices of the Republican period but recast them in the new paradigm of *bianzheng lunzhi*, as we will see with the treatment of emotion-related disorders.

## Coming Late to China

One of the surprising aspects of contemporary treatments for depression, with its strong focus on the Liver, is that this quintessentially “Chinese” approach to mental illness, locating emotion-related disorders in the body, was shaped to a considerable extent by the encounter with Western medicine. Although one might expect this influence to be relatively recent, a phenomenon of the contemporary period when Western medicine has become the dominant form of medical practice in China, it seems that the decisive moment of encounter was actually in the early twentieth century, prior to the arrival of modern psychiatry. Contemporary psychiatric disease categories, based on a biological understanding of mental disorders and treatable with certain psychoactive pharmaceuticals, were not codified in the West until 1952, when the *Diagnostical and Statistical Manual (DSM) of Mental Disorders* was first written. Because of China’s relative isolation from the (capitalist) West after the Communist Revolution in 1949 and the strong influence of Soviet neuropsychiatry in the 1950 and 1960s, the DSM psychiatric disease categories were not introduced into China until the 1980s (Lee 1999; Kleinman 1986, p. 33). As a result, doctors in China relied on older categories of neuropsychiatry, such as neurasthenia, which came to China in the 1920 and 1930s (see Kleinman 1986, pp. 25–29).

At just about the time neurasthenia was becoming known in China, it was falling out of favor in North America and Europe. By 1980, when DSM-III was published, it had been eliminated as a disease category, dismissed by its detractors as “a meaningless concept” (Kleinman 1986, p. 21). In spite of its ignominious decline, neurasthenia had once been an extremely important disease category in the West. George M. Beard first defined the term in 1869 as a functional disease of the nervous system, distinct from mental illnesses, such as insanity (Lee 1999). It had the appeal of identifying a putative site of illness for emotion-related disorders in a field of physiology that was developing rapidly. He noted that neurasthenia was most common during young adulthood or early middle age, exacerbated by the pressures of modern civilization and excessive mental labor, therefore making the educated most susceptible to this disease. By the late 19th century, Emile Durkheim commented that it had become a sign of distinction, constituting “almost a nobility” (Durkheim 1951 (1897), p. 181).

Neurasthenia came to China via Japan (Shapiro 2003). Known as *shikei suijaku* 神経衰弱 in Japanese and *shenjing shuairuo* 神經衰弱 in Chinese, this disease found a receptive population in Republican China, where the feeble, sickly individual was considered to be at the root of national weakness (Shapiro 1998). For doctors of Chinese medicine, neurasthenia was more than just a trendy new disease. It was also a vehicle for the concept of the nervous system as a whole, for which there was no comparable entity in Chinese medicine. As Hugh Shapiro has argued, Chinese medicine physicians had previously encountered Western medicine descriptions about the nervous system and its centrality to cognition and volition, but these physiological and philosophical claims had limited utility in the pragmatic world of Chinese medicine clinical practice. With the arrival of neurasthenia in the 1920 and 1930s, doctors of Chinese medicine then had an entry into thinking about nervous conditions in general (Shapiro 2003).

## Locating the Nerves in the Chinese Medicine Body

At this time, most doctors of Chinese medicine had no knowledge of biomedicine at all. Among those who did, an elite contingent of doctors interested in “reforming 改革” Chinese medicine began to reflect upon the significance of neurasthenia, nervous conditions, and the role of the nerves more broadly for the development of Chinese medicine. One of the interesting claims of this group was that the biomedical understanding of the nervous system could guide Chinese medicine treatments of emotion-related disorders because the nerves were roughly analogous to the Chinese medicine notion of the Liver. Zhang Cigong 章次公, a well-known reform-minded clinician and teacher of this period, demonstrates how this idea could be put into practice. In a book of his case records, he notes the relationship between the Liver, nerves and neurasthenia. Commenting on Mr. Liang’s insomnia, he says, “The ancients would consider this a case of Liver deficiency because the Liver stores the ethereal soul. Most herbs that tonify the Liver have the function of strengthening the nerves” (Zhu Liangchun 朱良春 1980, p. 229). In Mr. Zhou’s case, he illustrates how one might treat neurasthenia with Liver modulating herbs. Using a prescription in which seven out of eight total herbs directly or indirectly treat the Liver, he notes, “This formula is like a Chinese medicine tranquilizer, and it is appropriate for insomnia due to neurasthenia.” (Zhu Liangchun 朱良春 1980, p. 231).

What is most striking about these claims from a contemporary perspective is that today’s doctors of Chinese medicine would almost certainly reject this equivalence between the nerves and the Liver, even though they generally hold these Republican era innovators in extremely high regard. Contemporary doctors are undoubtedly much more knowledgeable about Western medicine then their Republican predecessors. The few Republican doctors of Chinese medicine who learned Western medicine were, for the most part, self-taught. By contrast, contemporary doctors have been receiving comprehensive training in biomedicine since the establishment of the state-run colleges and universities of Chinese medicine in the 1950 and 1960s because the curriculum at these schools has been more or less evenly split between courses in the two medical systems. Moreover, contemporary doctors have learned a vastly more advanced version of Western medicine, and they use it on a daily basis in their clinical work for the reasons described above. Yet, the “mistaken” claims of Republican era reformers, first asserted almost a century ago, continue to have an enduring influence on contemporary practice.

Before we examine how these ideas have been incorporated into contemporary practice, we need to understand why these early reformers believed the Liver in Chinese medicine was roughly analogous to the nervous system. How did they conclude that Liver therapies would be important for a range of neuropsychiatric conditions, from neurasthenia to dementias caused by stroke? One of the most cogent arguments was made by Lu Yuanlei 陸淵雷, a colleague of Zhang Cigong and a well-known advocate for “scientizing Chinese medicine 中醫科學化.” In his opus, *A Modern Interpretation of the Synopsis of the Golden Casket* 《金匮要略今释》, published in 1934, he reinterpreted the famous opening passage to this Han dynasty classic, *The Synopsis of the Golden Casket* 《金匮要略》 (ca. A.D. 200), to illustrate his new understanding of the Liver. The original passage reads as follow:

It is asked: How does the superior physician treat that which is not diseased? The master replies: in treating that which is not diseased, the physician must know that if he sees disease in the Liver, it will transmit to the Spleen, and he must first strengthen the Spleen…. The average physician does not understand the transmission of disease. When he sees Liver disease, he does not know to strengthen the Spleen and only treats the Liver (Lu Yuanlei 陸淵雷 2008 (1934), p. 2).

Classically, this passage has been interpreted as a statement of the importance of the Five Phases. The superior doctor must understand how the cycles of production and conquest in the Five Phases, such as Wood (the Liver) checking Earth (the Spleen), in order to prevent the transmission of disease. Lu Yuanlei’s commentary on this passage, however, begins with a dismissal of the Five Phases as empty speculation. He notes that this passage is stylistically different than the rest of *The* *Synopsis,* which otherwise rarely references Five Phases doctrine, demonstrating that it was not part of Zhang Zhongjing’s original text, but rather an addition by later commentators (Lu Yuanlei 陸淵雷 2008 (1934), p. 2). He nonetheless believes this spurious passage merits an explanation of Liver disease, Spleen disease and the mechanism of transmission from one to the other. As is so often the case with Lu Yuanlei, biomedicine provides the solid epistemological ground that he seeks:

What needs to be explored here is what kind of disease is Liver disease, what kind of disease is Spleen disease, and why must Liver disease transmit to the Spleen. To say that Liver Wood transmits to Spleen Earth and that Wood can check Earth is to use language that is empty and difficult to understand. According to the principles of the Inner Canon, the virtue of the Liver is joy and openness; the disease of the Liver is apprehension and brooding 鬱怒 [literally “constrained anger”]. What the ancient medical texts call the Liver for the most part refers to the nerves. With joy, the nerves are relaxed; with anxiety and anger, the nerves are excited…

The distribution and function of the sympathetic nervous system is for the purpose of fight or flight… As society evolved, humankind did not need to struggle with animals for food, the stimuli causing fear and anger gradually diminished while desires grew. Life became more complicated; some desires could not be fulfilled, leading to apprehension and brooding. But the human sympathetic nerves remained unchanged. Apprehension and brooding were sufficient to stimulate the sympathetic nerves and produce the usual reflexes. When apprehension and brooding ca not be relieved by the flight or fight response, the muscles are not used. As a result, the muscles have excess energy, and the meridians and blood vessels are stressed. If the brain has excess energy, then sleep is disturbed; if the heart and lungs have excess energy, panting and heart palpitations ensue. This is what the ancients called Liver disease. If the gastrointestinal track is inhibited, resulting in poor digestion, or dry heaves, or constipation, or stomach pain, this is what the ancients called the Liver transmitting to the Spleen (Lu Yuanlei 陸淵雷 2008 (1934), pp. 3–4).

The intellectual moves that Lu Yuanlei makes in this passage are not easy to follow. His re-interpretation of this passage from *The Synopsis* not only draws on the latest biomedical understandings of the nervous system, but also references Western discourses on evolution, social Darwinism, and civilization (Karl 2002; Duara 1995).^6^ His reformist colleague, Wang Yugao 王宇高, found this explanation unnecessarily convoluted and abstruse. In a letter to Lu Yuanlei, Wang Yugao argued that the Chinese medicine Liver affects the digestive system (both scholars concurred that the Chinese medicine Spleen could be equated to the biomedical digestive system) simply because modern physiology tells us that the anatomical liver produces bile, which empties into the small intestine to aid in digestion, but Lu Yuanlei rejected this apparently straightforward explanation. In a response later published in *The New Journal of Chinese Medicine* 《中醫新刊》, Lu Yuanlei argued that most classical passages on the Liver reference “the emotions of the brain 大腦之情緒” (Lu Yuanlei 陸淵雷 2008 (1934), pp. 77–78). In this era of rampant neurasthenia, emotion-related disorders were nervous disorders, and the cause of these nervous disorders for Lu Yuanlei was, above all, brooding 鬱怒 or, literally, “constrained anger.” Bringing together the pathology of constraint and the emotion (anger) classically associated with the Liver in this term, Lu Yuanlei argued that the unfulfilled desires of modern life excited or suppressed Liver function, leading not only to digestive problems, as the original passage tell us, but to a range of disorders.

Lu Yuanlei was a highly respected but extremely controversial figure in the Republican period, unafraid to challenge some of the most cherished concepts in Chinese medicine, always for the purpose of promoting his vision of Chinese medicine that was consistent with the standards of modern science. Although Lu Yuanlei’s attempts to integrate Chinese medicine and Western medicine were too radical for many in the profession, his views about the Liver and the nerves were widely shared among reformist doctors in Shanghai. For example, his teacher, Yun Tieqiao 惲鐵樵, argued in his textbook, *Exploring the Subtleties of the Pulse*《脉学发微》, that emotions, such as melancholy, anxiety and hatred, can stimulate the nerves and cause tension in the walls of the arteries, producing a wiry pulse 弦脉, the classic pulse associated with Liver pathologies.

Why is the pulse of the Liver wiry? Wiry indicates tension in the nerves of the arterial walls. Liver disease in *The Inner Canon* actually refers to brain disease. The classics usually associate anger with the Liver, which is why the Liver is the General, but in fact [the emotions of] the Liver include melancholy, hatred, neuroticism, and all the Seven Emotions 七情. Its diseases are integrally related to the brain and therefore are neurogenic (Yun Tieqiao 恽铁樵 2008, p. 48).

All diseases and symptoms that are related to the nerves should be discussed in terms of the Liver. Our generation cannot afford not to know this fact (Yun Tieqiao 恽铁樵 2008, p. 24).

Another famous Republican era physician, Zhu Weiju 祝味菊, known for his virtuoso clinical skills, also shared this understanding of the nervous system. Zhu Weiju had a strong background in Western medicine, having spent two years studying Western medicine at the Sichuan Military Medicine Academy and another year investigating medicine in Japan. In his 1931 book, *Elaborations on Pathology* 《病理发挥》, he argued that both Heart *qi* and Liver *qi* conditions in Chinese medicine refer to nervous system pathologies:

The more the world’s level of civilization progresses, the more knowledge advances, the more functional nervous disorders spread… The ancients were not good at anatomy and did not know what the nerves were. They speculated about the pathology of functional nervous disorders based on the symptoms, sometimes even attributing particular presentations to the various organs… References to all Heart *qi* and Liver *qi* diseases in the old medical texts correspond to what we today call nervous diseases. The correspondence is, in fact, quite good most of the time, just the names are different. Heart *qi* refers to functions of the voluntary nervous system; Liver *qi* refers to the functions of the autonomic nervous system. The ancient term “*qi*” encompasses all the functions of the nervous system (Zhu Weiju 祝味菊 2008, p. 14).

In contrast to Lu Yuanlei and Yun Tieqiao, Zhu Weiju’s discourse on Heart and Liver *qi* goes beyond emotion-related disorders to encompass all neurogenic conditions. Although he does not fully explain how the progress of civilization leads to nervous disorders, he seems to be invoking the same sociological claims of Lu Yuanlei: unfulfilled desires can either lead to a “suppression 痲痹” or “excitement 興奮” of nervous function.

These passages show that there was relative unanimity among these reformist voices; the Chinese medicine Liver was roughly analogous to the biomedical nervous system. Therefore Liver pathologies, like nervous pathologies, can arise from all kinds of emotional vicissitudes (not just anger as stated in the Chinese classics). These pathologies, which usually present as constraint, can be treated with Chinese medicine therapies. For reasons we will discuss below, these claims would be both commonplace and perplexing to the contemporary practitioner. The association of the Liver with emotional disruptions and constraint is a widely recognized feature of contemporary practice. But, this line of reasoning, in which the connection with the nervous system serves as the linchpin, would be unfamiliar, perhaps even shocking, to contemporary doctors of Chinese medicine. Today’s doctors are reluctant to equate the organs of the Chinese medicine body with those of the Western medicine body. They might assert that there is some conceptual overlap between the Chinese medicine Liver and the biomedical liver, but I have yet to encounter a text or an individual who reiterates the Republican era claims about the relationship between the Liver and the nerves.

## The Promise of Empiricism

What made these Republican Era reformers so confident about their claims and contemporary doctors so wary of them? One important difference between these two periods is that contemporary doctors perceive themselves to be heirs to a long, rich, unbroken tradition of medical scholarship. Republican Era reformers, however, were suspicious of their medical heritage. They championed the medical classics of early China, but were deeply skeptical about the previous millennium of medical writings beginning with the Song dynasty (960–1279). Another difference is that contemporary doctors have a complex relationship with Western medicine, simultaneously embracing and distancing themselves from this competing medical system (Karchmer 2010). Republican reformers, by contrast, embraced Western medicine much more fully, and saw it as an important tool to rectify the errors of late imperial medicine. The pioneer in this endeavor was Tang Zonghai with his work, *Essential Meanings of the Medical Classics in Light of the Convergence of East and West* 《中西汇通医经精义》, published in 1892.

Tang Zonghai and his followers admired the empiricism of Western medicine and attempted to use it to correct the speculative elements in Chinese medicine. Tang embarked upon this project because he believed that the two medical systems to be quite complementary, as the following passages from the “Prefatory Remarks 例言” of *Essential Meanings* shows:

Chinese drawings of the organs were all done in the Song and Yuan dynasties or later. They often do not correspond to the true structures of the human organs. Therefore all the drawings [in this book] are based on Western medicine drawings, which are more elegant than the old drawings.

I have used the anatomical drawings of Westerners but not their explanations. When verified against Inner Canon, the structures [in the drawings] are shown to be completely correct. If we use these drawings to seek the meaning of the Inner Canon, *qi* transformation becomes even more evident (Wang Mimi 王咪咪 and Li Lin 李林 1999, p. 4).

Tang Zhonghai identifies *qi* transformation as the unique characteristic of Chinese medicine, the feature of human life that anatomical dissections of corpses can never grasp. Yet it is the dissections of Western medicine, clearly superior to the erroneous Chinese ones, which can help illuminate the subtleties of *qi* transformation.

## The Critique of Speculation

These attitudes about Western science blended together with the evidential scholarship among late Qing literati to produce an even greater emphasis on empiricism in the late imperial and early Republican period. The work of the brilliant Zhang Taiyan 章太炎, who was one of the leading evidential scholars of his time, a major figure in the 1911 Revolution, an accomplished doctor in his own right and a mentor to many of the reformist doctors of the Republican period, offers a great example of this trend. Living in exile in Tokyo in 1911, on the eve of the Revolution, Zhang Taiyan wrote a letter to his disciple, Qian Xuantong 钱玄同, advising him how to proceed with his studies of medical texts. The sentiments expressed in the following passage exemplify the critical perspective of evidential scholars towards China’s medical heritage.

With medical writings, you can generally focus on those from the pre-Tang and most of the two Song dynasties. There is basically no need to look at the various writers from the Jin, Yuan, and Ming. You should peruse Yu Jiayan of the late Ming and Xu Zhongke and Ke Yunbo from the recent period [Qing]. The superficial writings of Ye Tianshi and Wu Jutong are not worthy of respect. The ancient pre-Tang texts are not more than ten. The *Divine Pivot* and *Simple Questions* are indeed the foundations. Put your emphasis on the pathways of the meridians and the transmission of disease. You can skip the strained passages pertaining to the Five Phases. Although *The Classic of Eighty*-*one Difficulties* is an ancient text, it contains a great deal of exaggerated language. No more than one to two tenths is valuable. Only *The Treatise on Cold Damage* and *The Synopsis of the Golden Chamber* approach the Way. With language that is comprehensive and precise, these texts rarely use the strained discourse of the Five Phases, relying only on the [patient’s] presentation to craft a formula. But its formulas are limited, so consult formulas from *Formulas Worth a Thousand Pieces of Gold* and *The Essential Secrets of the Outer Terrace* (which contain numerous formulas from the Six Dynasties era). These two texts have many formulas but too little discussion of disease. If you do not first understand *The Treatise* and *The Synopsis*, then it is not possible to use their formulas well. You should also peruse works from the Song dynasty, such as Zhu Gong’s *Formulas to Save Lives* 《活人書》,… *Good Formulas of Su and Shen* 《蘇沈良方》,…Xu Shuwei’s *Formulas of Ability* 《本事方》, and the state sponsored texts of the time, such as *The General Record of Sacred Assistance* 《聖濟總錄》 (from the Huizong reign) and *Formulas for Benevolent Assistance* 《和劑局方》 (Zhang Taiyan 章太炎 2009, p. 222).

In this passage, Zhang Taiyan illustrates the classic intellectual stance of evidential scholarship, celebrating (most) Han and pre-Han medical texts and expressing deep skepticism about of the value post-Song medical writings (Elman 1984). The Han dynasty writings of Zhang Zhongjing, as found in *The Treatise on Cold Damage* and *The Synopsis of the Golden Chamber*, are the pinnacle of medical scholarship not only because their antiquity, but also because of their empiricism. For Zhang Taiyan, the empiricism of these texts is found in their careful avoidance of speculative philosophy, such as the Five Phases, and their reliance on the principle of “examining the presentation to craft a formula 審證處方.”^7^ Although the importance of evidential scholarship would begin to decline with the fall of Qing dynasty and the end of the imperial examination system, many of the Republican era reformers were not only steeped in it from their early education, but also directly influenced by Zhang Taiyan, whom they revered as teacher and statesman.

One important consequence of the influence of evidential scholarship, especially in the Shanghai-based reformers, was a deep antipathy towards the Warm Illness current, a major development in medical discourse originating in the Jiangnan region during the Ming and Qing dynasties. Zhang Taiyan’s sweeping dismissal of the two key innovators in the Warm Illness current, Ye Tianshi and Wu Jutong, was a common refrain in their writings. Although most historians of the Republican Era have focused on the debates between the Western medicine and Chinese medicine professions, the tensions between the Warm Illness and Cold Damage camps may have been even more divisive (See Croizier 1968; Lei and Sean Hsiang-Lin 1999; Zhao Hongjun 赵洪钧 1982). In his 1932 text, *A Collection* *of Insights from Discussing Medicine in the Reading Room* 《籀簃谈医一得集》, Zhang Shanlei 張山雷 offers one typical example of the heated rhetoric used by the Cold Damage proponents to attack the Warm Illnesses current.

When the warm heat theory of Ye Xiangyan [Tianshi] became popular, there was the possibility that some of the inspirations of later scholars could slightly augment that which was missing in Zhang Zhongjing’s *Treatise*. But who could have imagined that Old Ye [Tianshi], the first to propose this theory, and [Wu] Jutong, the first to write a treatise about it, would both shun Zhang Zhongjing’s established principles, erroneously creating new formulas, using cloying herbs that trap the pathogen, causing innumerable harms without a single benefit. Everyone has followed in this path without reflection, adopting habits that completely mislead the people, devoting one’s entire life [to this mistaken approach] without ever awakening (Zhang Shouyi 张寿颐 2008, p. 78).

Yun Tieqiao and Lu Yuanlei also made no secret of their distaste for the Warm Illness current and its leading figures, Ye Tianshi, Wu Jutong and Wang Shixiong, and frequently described their writings as a “flowing poison 流毒.” This animosity would dissipate fairly quickly in the Communist Era, but during the Republican period, reformist scholars felt the Warm Illness current was so pernicious that it made the reform of Chinese medicine the only hope for the survival of Chinese medicine. As Volker Scheid’s research helps illustrate, one of the ironies of this rejection of Warm Illness doctrine was that Republican Era reformers quietly embraced many other practices associated with Ye Tianshi. In particular, their understanding of Liver constraint and its relationship to the emotions closely followed Ye Tianshi’s innovations, even though they did not acknowledge and may not have even been aware of this debt to him (Scheid 2013).

## The Example of Japan

The rise of Japan as new power in East Asia added further complexities to these debates, as different observers drew different lessons from the rapid pace of Japan’s modernization. One lesson was to emulate Japan’s radical promotion of Western medicine over traditional medicine. Following the Meiji Restoration in 1868, the Japanese state sought to explicitly follow a Western model of development, which included the active promotion of Western medicine over traditional Kampo medicine. By the early 1900s, and especially after the defeat of Russia in the Russo-Japanese War of 1905, Japan could claim to be a world power. This sudden rise to dominance in East Asia made Japan the most important destination in the early twentieth century for young Chinese students eager to study the latest in “Western learning 西學”, including Western medicine. Japan’s medical policy made a strong impression on some of these students, including Yu Yunxiu 余雲岫. Originally trained as a Chinese medicine doctor before he departed for Japan to study Western medicine, Yu Yunxiu returned to China in 1916 to become one of the most outspoken critics of Chinese medicine. He argued that Chinese medicine, especially its speculative theories about the human body, was clouding the minds of average citizens and thereby impeding the modernization of the nation. He became famous for his attempt to ban Chinese medicine in 1929 as a member of the Ministry of Health (Croizier 1968; Deng Tietao 邓铁涛 1999; Lei 1999).

Practitioners of Chinese medicine took a very different lesson from Japan’s medical policies. Some perceived the banning of Kampo medicine in Japan as a failed policy that Chinese government officials must avoid. Shortly after Yu Yunxiu’s proposed bill to ban Chinese medicine, Zhang Xichun 張錫純, the famous reformist doctor from Tianjin, argued this point, using case studies from Japan to demonstrate the decline of clinical medicine in Japan (Zhang Xichun 张锡纯 1929). This argument would have found a receptive audience because it was generally accepted that Chinese medicine was the clinically more effective form of medicine, even if it was less “scientific”.^8^ For doctors of Chinese medicine, the perceived clinical advantages of Chinese medicine were also confirmed by the 1920’s revival of Kampo medicine in Japan. Yumoto Kyushin 湯本求真, a Western medicine doctor who had turned to the Ancient Formulas current of Yoshimasu Todo when he was unable to prevent the deaths of his own children, became one of the leading figures in this movement. His work, *Sino*-*Japanese Medicine* 《皇漢醫學》, published in 1927 (and translated into Chinese in 1928), was widely read in China and inspired the publication of a collection of 72 Japanese medical texts, *The Sino*-*Japanese Medical Series* 《皇漢醫學叢書》, in 1936. Some of the leading contemporary doctors in China, such as Yu Yibai 于已百 of Lanzhou and Liu Shaowu 劉紹武 of Shanxi, attribute their success to the influence of these Japanese texts (Liu Shaowu 刘绍武 and Liu Huisheng 刘惠生 2002).^9^ More recently, now that post-war animosity towards Japan has waned, Huang Huang 黃煌, professor at Nanjing University of Chinese Medicine, has rediscovered the excitement of Japanese Kampo scholarship and is sharing it with a new audience of young Chinese medicine doctors.

The Japanese revival of Kampo medicine provided further impetus to the empiricist proclivities of the Chinese medicine reformers in Shanghai. As Daidoji explains, the Ancient Formula current *kohoha* 古方派 formed the basis for the twentieth century revival of Kampo medicine (Daidoji 2013). Its iconic progenitor, Yoshimasu Todo 吉益東洞, considered Zhang Zhongjing as the ultimate source of medical knowledge and spurned all speculation about disease pathology and the inner workings of the body. His clinical style relied exclusively on Zhang Zhongjing’s work, matching symptoms directly to formula. The discovery of this current of Japanese medical writings generated great enthusiasm among some reformist doctors in China, such as Lu Yuanlei.

I’ve recently read the works of the Eastern doctor, Yoshimasu Todo, such as *Formulas by Categories* ≪类聚方≫ and *Formula Standards and Medicinal Evidence* ≪方极药征≫, which coincidentally match my views. Treating diseases according to his recommendations is very effective. Moreover, Todo is a minimalist. He not only rejects the Five Evolutive Phases and the Six Climatic Factors, but also dismisses all the commentary on disease names in Zhang Zhongjing’s works. He was not mistaken in this position, knowing exactly where the essence of Chinese medicine lies (Lu Yuanlei 陸淵雷 2008, p. 83).

Prior to the late nineteenth century, Japanese scholarship on East Asian medicine was almost unknown in China (Mayanagi Makota 真柳誠 2011). During the growing political entanglement and cultural exchange of the early modern period, it became quite influential among reformist scholars in Shanghai. The strong empiricist emphasis in Japanese Kampo medicine, especially the Ancient Formulas current, made a perfect synergy with the lessons reformists were drawing from Western medicine and Confucian evidential scholarship. At the Shanghai China Medical College, where Lu Yuanlei, Zhang Cigong, and other reformists taught, there was a general appreciation for Japanese medical scholarship. In the first year textbook, General Medical Knowledge 《醫學常識》, written by Zhang Henian 章鶴年 students are presented with a list of 39 must-read books for their profession and 11 of them are by Japanese authors.^10^ Outside of Shanghai, however, there was skepticism towards Japanese medical scholarship. In an open debate with Yumoto Kyushin 湯本求真, one of the editors of the Tianjin-based journal, *The Righteous Words of National Medicine* 《國醫正言》, Wu Hanxian 吳漢僊 expressed incredulity at Yumoto Kyushin’s insistence on using only Zhang Zhongjing formulas for all conditions, including those not explicitly addressed in *The Treatise* or *The Synopsis* (Wu Hanxian 吳漢僊 1935).^11^

## Refashioning the Nation

The three intellectual streams that were driving reformist scholarship in the Republican period lost their significance or were altered by WWII and the Communist Revolution. For younger doctors born in the Republican period, the importance of Confucian evidential scholarship was fading rapidly. Interviews with surviving doctors from this era confirm that the Confucian classics remained the foundation of their early education in the countryside, but urban youth were increasingly exposed to modern primary and secondary education before beginning their medical studies.^12^ Following the Communist Revolution, Marxism and dialectical materialism became the only philosophical system that was allowed by the state, and young doctors were encouraged to participate in short courses or study on their own. Some doctors found much inspirational material within the Marxist corpus. For example, Li Zhenhua’s understanding of *The Inner Canon* was transformed by his encounter with Engel’s *Dialectics of Nature* 自然辩证法, which helped him realize that Chinese medicine had its own “simple materialism 朴素唯物主义”.^13^ With a new grasp of dialectical materialism, scholars began to see the history of Chinese medicine from an evolutionary perspective. Progress, not regression, defined China’s long history of medicine. It was no longer possible to dismiss the entire post-Song millennium of medical scholarship as empty speculation and decline.

In the 1950s, nationalism and national unity took precedence over the cultural iconoclasm of the Republican period. Social critique did not disappear, indeed it became much more violent, but it was re-channeled into Marxist class struggle, which had less direct impact on doctors of Chinese medicine than it did on other groups. Moreover, the Communist party called for unity between the Chinese medicine and Western medicine professions 中西醫團結 and took direct measures to curtail official bias towards Chinese medicine from 1954 on (Taylor 2004; Lampton 1977). It is not surprising that following the war with Japan, there was little interest in promoting scholarly exchange with Japan. As a result, the influence of Japanese medical scholarship was much smaller for the next generation. Perhaps without this international influence, Chinese medicine became even more “national” in its focus. Divisiveness, such as the bitter animosity between Cold Damage and Warm Illness, was frowned upon. Scholars, such as Deng Tietao 鄧鐵濤, saw the need to heal these wounds in the mid 1950s. Deng argued in an influential article in *The Journal of Chinese Medicine* that Warm Illness scholarship built upon and developed Cold Damage scholarship, that wholesale denunciations of one school by another contradicted the principle of historical development. His paper was well received by his peers and has now become the conventional wisdom (Deng Tietao 鄧鐵濤 1955).

The most important change for the Chinese medicine profession was its new relationship with Western medicine. In spite of heated political rhetoric during the Republican period, doctors of Chinese medicine perceived the epistemological divide between the two medical practices to be relatively small. Reformists felt free to borrow theories, concepts, techniques, and drugs from Western medicine to deepen their understanding of their own medical practice. This relationship was facilitated by the numerical preponderance of Chinese medicine doctors and the perception that Chinese medicine was the clinically superior form of medicine, in spite of its less than “scientific” status. During the Communist period, this sense of superiority dissipated; the two types of medicine seemingly grew apart epistemologically, even as their political cooperation increased. In the Republican period, reformists considered it enlightening and forward thinking to explain the Chinese medicine Liver in terms of the nerves; in the Communist era, these kinds of comparisons have been viewed as crude and even potentially dangerous.

Today’s doctors assert that their caution towards making equivalences between Chinese medicine and Western medicine is due to their more comprehensive knowledge of Western medicine than their Republican predecessors. But these new sensitivities are probably also a response to the demographic changes that have profoundly altered the terrain of medical practice in China after the Communist Revolution. Although Communist party policy has supported the Chinese medicine profession, it has devoted far greater resources to building the Western medicine profession. According to official Ministry of Health statistics, in 1949 there were at least ten times the number of Chinese medicine doctors as Western medicine doctors. Today the latter is five times more than the former (Editorial Committee of the China Medical Yearbook 《中國衛生年鑒》編輯委員會 2001, p. 455). The second half of the twentieth century is also the period when the biomedical profession made some of its most dramatic technological and therapeutic advances. As doctors of Chinese medicine became aware of their relative weaknesses—politically, socially, and clinically—they sought to define their uniqueness.

## Textbook Medicine

These new pressures were the background to one of the most important achievements of the Communist Era: the creation of a set of national textbooks that defined standard terms, concepts, diagnoses and therapies for the profession. In 1956, the Chinese state established the first four colleges of Chinese medicine—in Beijing, Shanghai, Guangzhou and Chengdu—and soon followed with the founding of colleges in most other provincial capitals. Professors from these new colleges were brought together in 1960 to collectively edit the first set of national textbooks, and again in 1963–1964 to create the authoritative second edition, which became something of a template for all future editions. The achievement of these editors should not be underestimated. The national textbooks not only became the foundation of the modern education system for Chinese medicine in China, but they have been translated into multiple languages, making possible the globalization of Chinese medicine today.

The collective textbook editing process produced a new medical discourse that emphasized editorial consensus and the uniqueness of Chinese medicine. As we will see through an examination of the illness of Constraint in the first and second edition of the national textbooks, Communist Era textbook editors did not reject Republican innovation, rather they incorporated it into what became the new paradigm of clinical practice: “pattern recognition and treatment determination *bianzheng lunzhi*” (Farquhar 1994; Scheid 2002). Because *bianzheng lunzhi* is considered such an integral part of contemporary Chinese medicine practice, doctors frequently use this term broadly as the key marker of good clinical practice. In my discussion, I will focus on the standard textbook definition of this methodology, which not only forms the basis of the education system, but has also become enormously influential in contemporary research and scholarship. What I hope to demonstrate here is that the Republican Era proclivities for seeking correspondences between specific features of Chinese medicine and Western medicine were replaced by *bianzheng lunzhi*, which emphasizes the distinctions between the two medical systems but also provides a means for integrating them (see Karchmer 2010).

In the 1960 first edition of *Chinese Internal Medicine* textbook, the insights of the Republican reformists concerning the illness of Constraint were recast in the language of a “pure” Chinese medicine. The editors emphasized the centrality of the Liver, the pathology of constraint, and the emotion-related etiology of this illness. But instead of explaining this condition through the operations of the nervous system, the editors derived it from the Yuan dynasty master, Zhu Danxi 朱丹溪:

Constraint is an illness caused by emotional thwarting. Zhu Danxi stated: “When blood and *qi* flow freely, no disease can arise. When they are impeded, all diseases are possible.” On the basis of this view, he developed the doctrine of six constraints, and among these six he clearly identified disease arising from *qi* constraint, which in turn caused other forms of constraints, such as dampness, phlegm, blood, food, etc. Constraint usually begins with Liver *qi* constraint… (Internal Medicine Department of the Shanghai College of Chinese Medicine 上海中醫學院內科教研組 1960, pp. 143–144)

This passage provides an excellent example of the process of consensus at work. As Volker Scheid has demonstrated, the emotions and the Liver were only tangential concerns in Zhu Danxi’s discourse on constraint (Scheid 2013). By invoking Zhu Danxi, the editors produced an illness category that seemed to draw on the long heritage of Chinese medical scholarship. But the discussion on treatment corroborates that it was their Republican Era predecessors, whose views were too divisive and too recent to be mentioned directly in the textbook, who most inspired their conclusions. Five out of the six recommended treatment principles focused on treating the Liver directly.^14^

In the authoritative second edition of the same textbook, the editors continued to stress the role of the emotions and the Liver in the pathology of Constraint:

The reason for Constraint always involves injury by the Seven Emotions 七情, which can in turn disrupt the *qi* mechanism of the five organs…. It’s pathomechanism is as follows: 1) Brooding [literally “constrained anger 鬱怒”] cannot be relieved. Liver wood cannot extend; *qi* does not flow and becomes unruly. It might disrupt the spirit of the Heart, attack the Spleen and Stomach, insult Lung metal, harass the meridians, or enter the intestines, causing all kinds of illnesses. 2) Continuous worrying and an unrelieved sense of being wronged may cause Liver constraint to reach the Spleen. Spleen transformation falters and phlegm arises… (Shanghai College of Chinese Medicine 上海中醫學院內科教研組 1964, p. 336).

Like the first edition, this explanation is justified through reference to ancient authorities, such as the *Inner Canon*, Zhu Danxi, and others. But the focus on Liver constraint and the role of the emotions suggests the greatest influence came from Republican Era reformers, the reference to “brooding 鬱怒” as a cause of Liver constraint hinting that Lu Yuanlei may have directly inspired this passage. The major difference with the first edition can be found in the treatment section. The second edition textbooks are the first ones to explicitly use *bianzheng lunzhi* as its organizing principles, which means that *bing* are now subdivided into multiple patterns *zheng* (Deng Zhongguang 邓中光, Zheng Hong 郑洪, and Chen Anlin 陈安琳 2004, p. 142). The editors listed five patterns, four of them corresponding exactly to ones identified by Zhang Yanhua in her research, the fifth one being something of a combination of the other two she reports. The first pattern in the textbook was, not surprisingly, Liver *qi* constraint, suggesting that this pattern was the most important one.

The second edition *Chinese Internal Medicine* textbook did not discuss Western medicine. But with the introduction of *bianzheng lunzhi*, the editors had, unwittingly perhaps, created an effective new mechanism for integrating Chinese medicine with Western medicine. Moving away from the physiological equivalences of the Republican era but keeping the insights from associating the nerves with the Liver, the formulators of *bianzheng lunzhi* asserted that the diagnostic processes of the two medical systems take place in different registers, one centered on the concept of disease, the other on the concept of pattern. But *bianzheng lunzhi* also made it possible to develop Chinese medicine treatments for biomedical conditions, by establishing an equivalence between a disease and a *bing.* Thus, if depression is roughly equivalent to Constraint, then it can be treated according to the standard patterns now associated with this *bing*. Zhou Ling and Tang Qisheng, cited at the beginning of this paper, show how contemporary researchers rely on this technique of integration and can adapt to their own purposes. One important advantage of *bianzheng lunzhi* is that it has promoted the globalization of Chinese medicine by transforming the concept of *zheng* 證, which used to loosely mean “symptom” in the early twentieth century into the new diagnostic category of “pattern”. Because patterns do not compete with biomedical diseases, but rather compliment them nicely, patterns can travel easily. As Volker Scheid and Soyoung Suh have shown, new East Asian *bing*/diseases, such as Constraint or *Hwa*-*byung* have struggled to secure a foothold in world of biomedicine (Scheid 2013; Suh 2013). But Liver *qi* constraint and other patterns have spread rapidly along alternative medicine networks, sometimes leading to their uncritical overuse in the care of emotion-related disorders. Many practitioners embrace these patterns precisely because they seem to offer alternatives to biomedicine. If they were aware of their entangled relationship with biomedicine, they might use them with more caution.

## Footnotes

1 In this paper, I use the term “Chinese medicine,” a direct translation of *zhongyi* 中医, the official designation for indigenous Han medicine in China. (Minority groups have different designations for their medical practices.) Until recently, most literature published outside of China has used the term “traditional Chinese medicine,” which was first promoted by the Chinese state in its English language publications in the late 1950s (see Taylor 2004). I prefer the term “Chinese medicine” because it is emphasizes the dialogic relationship of this practice to “Western medicine 西医,” which is the official designation for biomedicine in China.

2 In order to better understand strategies of integration, I will leave the Chinese medicine notion of *bing* 病 untranslated to distinguish it from the biomedical concept of disease, which is expressed by the very same Chinese term, *bing* 病. Doctors of Chinese medicine sometimes equate *bing* and disease in practice, but most doctors recognize these correspondences as partial and limited.

3 This convention developed in the late 1950 and early 1960s, when state-run Chinese medicine hospitals were first being established. It is an obligatory and central part of all medical records in a Chinese medicine hospital (see Karchmer 2010).

4 Because Chinese medicine and biomedicine have very different understandings of the organs, I capitalize the former to distinguish it from the latter.

5 National standards for Chinese medicine diagnosis were first issued in 1995. The pattern subcategorization for constraint illness in this clinical reference is fairly close to the patterns Zhang observes, recommending five patterns instead of six. They are Liver *qi* constraint, Qi constraint transforming in fire, constrained anxiety injuring the Spirit, Heart and Spleen Deficiency, Deficient Yin and blazing fire. Four of them match exactly the patterns that Zhang reports (State Administration of Traditional Chinese Medicine 國家中醫藥管理局 1994).

6 Yan Fu’s translation of Thomas Henry Huxley’s *Evolution and Ethics* first introduced social Darwinist discourse in China in 1895 (Schwartz 1964). The notion that races and nations were subject to evolutionary pressures and “the survival of the fittest” became very influential among early twentieth century Chinese intellectuals (Pusey and James Reeve 1983). At the same time, Chinese intellectuals were embracing and modifying the European discourse of civilization that was so often used to justify imperialist encroachments (Duara 2003, pp. 89–129). Lu Yuanlei’s familiarity with these debates, his detailed knowledge of Western medicine, and his mastery of the classic medical texts is part of what made him such an impressive scholar within the Chinese medicine community.

7 The term that I have translated as “presentation” here is *zheng* 證. After the 1950s, it will acquire a very different connotation, which is now usually translated as “pattern.” I will discuss this shift in meaning briefly at the end of the paper. (For additional discussion, see Karchmer 2010.)

8 Lu Yuanlei gives an example of this assumption of clinical superiority. Commenting on the treatment of infectious diseases (prior to the discovery of antibiotics), Lu Yuanlei stated, “Nine out of ten Western medicine treatments for infectious diseases are ineffective… When treating infectious diseases, Chinese medicine can truly help the patient resist the infection. But there is not nearly enough space here [in this journal] for me to explain the principle. If we ban Chinese medicine and replace it with Western medicine now, then we will be even less able to treat infectious diseases (Lu Yuanlei 陸淵雷 2008, pp. 87–88)!”

9 Personal interview, Yu Yibai 于已百, March 28, 2009.

10 Interest in Japanese medicine declined sharply with the hostilities of the Sino-Japanese War and World War II. When I was studying Chinese medicine at the Beijing University of Chinese Medicine in the late 1990 s, there was very little interest in Kampo medicine at my university, and I was not encouraged to read any medical texts from Japan. From time to time, I would hear my teachers speak dismissively of Kampo medicine, as if it were a simplified, derivative version of Chinese medicine. Since the early 2000’s, however, numerous Kampo texts have been published in China, suggesting a renewed interest in this scholarship.

11 In his debate, Wu Hanxian referred to a division between internal and external diseases that dates back to the Ming dynasty (1368–1644). Asserting that *Treatise* formulas were clearly designed for treating externally contracted diseases only, Wu Hanxian asked Yumoto if he would still insist on using them to treat a case of internal damage with fever. The famous Jin dynasty doctor Li Dongyuan had created a formula, Tonify the Center and Supplement Qi Decoction 補中益氣湯, precisely for this condition. Staying true to his principles and rejecting the Ming division of internal and external diseases, Yumoto Kyushin responded it was better to use Small Bupleurum Decoction 小柴胡湯 with Angelica and Peony Powder 黨歸芍藥散 (two formulas from Zhang Zhongjing’s works) than Li Dongyuan’s formula.

12 Personal interviews: Wang Jialin 王嘉麟 (June 2011), Li Jinyong 李今庸 (July 2011), Zhu Liangchun 朱良春 (December 2008).

13 Personal interview: Li Zhenhua 李振華 (March, 2011).

14 In 1960, the concept of *bianzheng lunzhi* was just emerging, and the practice of subdividing a *bing* into multiple patterns, each with an indicated formula, had not yet emerged. Instead the editors proposed six different treatment principles, recommending several possible formulas for each. Five of the treatment principles involve the Liver: dredging 疏, draining 泄, leveling 平, clearing 清, and softening 柔 the Liver; the sixth method activates the blood, including several herbs with affinities for the Liver.
